# Direct, Indirect, and Intangible Costs in the Management of Dental Caries in Children Under 18: A Systematic Review

**DOI:** 10.3390/dj14090578

**Published:** 2026-09-09

**Authors:** Darja Gostilo, Xenia Teplitzky, Blend Hamza, Georgios Tsilingaridis, Sivaprakash Rajasekharan, Līna Petrova, Megija Florentine, Shaju Jacob Pulikkotil

**Affiliations:** 1 Prof. Shaju Jacob Pulikkotil Group, Oral Health Research, Faculty of Dentistry, Rīga Stradiņš University, LV-1007 Riga, Latvia; xenia.teplitzky@rsu.lv (X.T.); lina.petrova@rsu.lv (L.P.); megija.florentine@rsu.lv (M.F.); 2 Clinic of Orthodontics and Pediatric Dentistry, Center for Dental Medicine, University of Zurich, Plattenstrasse 11, 8032 Zurich, Switzerland; blend.hamza@zzm.uzh.ch; 3 Division of Pediatric Dentistry, Department of Dental Medicine, Karolinska Institutet, 171 77 Stockholm, Sweden; georgios.tsilingaridis@ki.se; 4 ELOHA (Equal Lifelong Oral Health for All) Research Group, Paediatric Dentistry, Oral Health Sciences, Ghent University Hospital, 9000 Ghent, Belgium; sivaprakash.rajasekharan@ugent.be

**Keywords:** dental caries, paediatric population, economic burden, cost analysis, preventive oral health

## Abstract

**Background/Objectives**: Dental caries remains one of the most prevalent chronic diseases affecting children worldwide and continues to pose a substantial challenge to healthcare systems. Although the clinical consequences of paediatric dental caries are well documented, the associated economic burden has not been comprehensively synthesised. This systematic review aimed to evaluate and summarise the available evidence regarding the costs associated with dental caries in children and adolescents under 18 years of age. **Methods**: A systematic review was conducted in accordance with PRISMA 2020 guidelines and registered in PROSPERO (CRD420251138979). MEDLINE via PubMed, Scopus, and Dentistry & Oral Sciences Source (EBSCOhost) were searched for studies published between January 2012 and July 2025. The MEDLINE and Scopus searches were conducted on 22 July 2025, and an additional search of Dentistry & Oral Sciences Source (EBSCOhost) was conducted on 19 August 2026. Eligible studies included peer-reviewed cost-of-illness studies, economic evaluations, cost analyses, cross-sectional studies, retrospective studies, and randomised controlled trials reporting economic outcomes related to the prevention or management of dental caries in individuals under 18 years of age. Methodological quality was assessed using the Drummond 10-item checklist. Data were extracted on study characteristics, population demographics, cost components, study perspective, and key findings. Reported costs were categorised as direct, indirect, and intangible costs where applicable. Due to substantial methodological heterogeneity among studies, a narrative synthesis was performed. **Results**: Seventeen studies met the inclusion criteria. Most studies focused on direct medical costs associated with restorative treatment, hospital-based dental care, and emergency services. Direct costs were consistently substantial across different healthcare settings, particularly for cases requiring advanced treatment or general anaesthesia. Preventive interventions were generally reported to be more cost-effective than curative approaches. Indirect costs, including caregiver productivity losses, were infrequently assessed, while no study comprehensively evaluated intangible costs. Considerable variation in study design, costing perspective, and reporting methodology limited direct comparison across studies. **Conclusions**: Dental caries in individuals under 18 years of age imposes a considerable economic burden, driven primarily by direct treatment costs. Preventive oral health strategies appear to provide greater economic value than treatment-focused approaches. The limited assessment of indirect and intangible costs suggests that the overall societal burden of paediatric dental caries is likely underestimated. Future research should adopt standardised and comprehensive costing methodologies to better support policy development and resource allocation in paediatric oral healthcare.

## 1. Introduction

Dental caries in primary and permanent teeth remains the most prevalent chronic disease among children worldwide and represents a major public health challenge [1,2]. Despite being largely preventable, untreated dental caries continues to affect millions of children, resulting in pain, infection, impaired oral function, and reduced quality of life [3]. The Global Burden of Disease (GBD) study has consistently identified untreated caries as one of the most common health conditions globally, highlighting its substantial impact on both individual well-being and healthcare systems [3].

The development of dental caries is influenced by a complex interaction of biological, behavioural, and socioeconomic factors. Poor oral hygiene, frequent consumption of free sugars, inadequate fluoride exposure, and limited access to preventive dental services are among the most important risk factors associated with disease progression [1,4]. In severe cases, dental caries may require extensive restorative treatment, hospitalisation, or treatment under general anaesthesia, resulting in significantly higher healthcare expenditures compared with preventive or routine dental care [1,4].

The burden of paediatric dental caries is particularly evident in Europe, where substantial inequalities in oral health outcomes persist. Although several Western European countries have achieved relatively low levels of caries experience, children living in Eastern and lower-income European countries continue to experience disproportionately higher disease prevalence [5]. For example, studies from Latvia have reported exceptionally high levels of dental caries among children, with nearly all 12-year-olds exhibiting signs of caries experience according to D1MFT criteria [6]. Furthermore, the World Health Organization estimated that 47.5% of Latvian children aged 1–9 years had untreated caries in primary teeth in 2019 [7]. These findings reflect a broader regional trend and suggest ongoing challenges in the implementation of effective preventive strategies.

Beyond its clinical consequences, dental caries impose a substantial economic burden on families, healthcare systems, and society [8]. Direct costs include expenditures associated with diagnosis, treatment, hospital-based care, and preventive services. Indirect costs arise from productivity losses experienced by caregivers who must take time away from work to attend dental appointments or care for affected children. In addition, intangible costs, such as pain, anxiety, reduced quality of life, and psychosocial distress, may considerably affect children and their families but are rarely quantified in economic assessments [8,9]. Evidence from other health conditions suggests that these intangible consequences can represent an important component of the overall disease burden and should be considered alongside direct and indirect costs [9].

To comprehensively assess the societal and economic implications of dental caries in children under 18, researchers increasingly adopt cost-effectiveness analyses using health outcome measures such as quality-adjusted life years (QALYs) and disability-adjusted life years (DALYs) [8]. Moreover, insights from other disease areas, such as cancer research, suggest the importance of accounting for intangible burdens, including psychological distress, financial anxiety, and coping behaviours, that shape health outcomes and care-seeking practices [9].

This systematic review aims to explore the available international evidence on the direct medical, non-medical, indirect, and intangible costs associated with managing dental caries in children under 18 years, to understand which solutions have been applied in other countries and to assess which of these approaches may be most beneficial for European healthcare systems.

Previous systematic reviews have primarily focused on clinical effectiveness and cost-effectiveness of early caries treatment and prevention or calculated costs of a specific treatment method rather than providing analysis of the economic burden associated with the disease [10].

Systematic reviews analysing evidence on indirect and intangible costs of paediatric dental caries are scarce and, to our knowledge, no systematic review has integrated direct, indirect, and intangible costs into a single review.

## 2. Materials and Methods

### 2.1. Search Strategy

This systematic review was conducted in accordance with the PRISMA 2020 reporting guideline, as Supplementary File S1: PRISMA 2020 checklist [11]. The review protocol was registered in the International Prospective Register of Systematic Reviews (PROSPERO; registration number CRD420251138979).

A systematic search of the peer-reviewed literature was conducted in MEDLINE via PubMed, Scopus, and Dentistry & Oral Sciences Source (EBSCOhost). These databases were selected to provide complementary coverage of the biomedical, dental, public health, and interdisciplinary health economic literature. MEDLINE provides focused coverage of biomedical and dental research, Scopus offers broad multidisciplinary coverage, and Dentistry & Oral Sciences Source (EBSCOhost) was additionally searched to broaden retrieval and reduce the likelihood of missing relevant economic evaluations. The MEDLINE and Scopus searches covered publications from January 2012 to July 2025 and were conducted on 22 July 2025. An additional search of Dentistry & Oral Sciences Source (EBSCOhost), applying the same eligibility period and conceptually equivalent search terms adapted to its indexing structure, was conducted on 19 August 2026.

Search terms combined controlled vocabulary, where available, and free-text terms related to dental caries and economic burden, including direct, indirect, non-medical and intangible costs, caregiver burden, school absenteeism, productivity losses, and health economic outcomes. The search syntax was adapted to the indexing system and field structure of each database. The complete database-specific search strategies, applied limits, search dates, and numbers of records retrieved are provided in Supplementary Table S5.

In addition to the database searches, backward citation searching was performed by manually screening the reference lists of all included studies and relevant systematic reviews and meta-analyses to identify potentially eligible studies that had not been captured by the electronic searches. Grey literature sources were not searched systematically.

**Figure 1:**
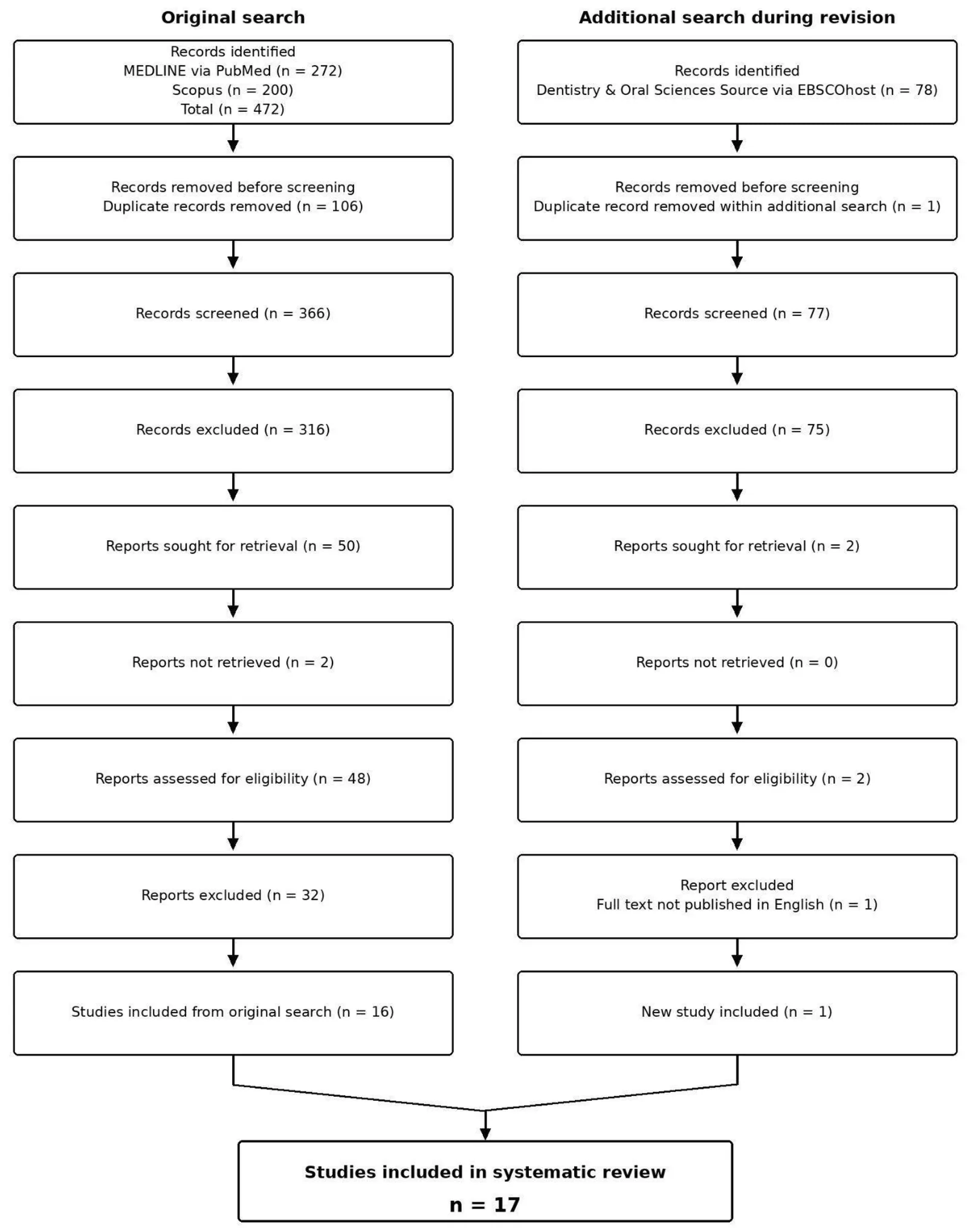
PRISMA 2020 flow diagram of study identification, screening, eligibility assessment, and inclusion, including the additional database search conducted during revision.

All retrieved records were imported into EndNote 21 (Clarivate, Philadelphia, PA, USA), and duplicate records were removed before screening. Reasons for exclusion at the full-text stage were recorded and are summarised in the PRISMA flow diagram (Figure 1).

### 2.2. Study Selection

Two reviewers (DG, SJP) screened the titles and abstracts of all retrieved records against the predefined eligibility criteria. Two reviewers (DG, SJP) subsequently assessed the full texts of potentially eligible studies. No automation tools were used during study selection.

### 2.3. Inclusion and Exclusion Criteria

The inclusion and exclusion criteria were guided by the PICOS framework from the review protocol:

Population (P): Children and adolescents under 18 years with dental caries or at risk of caries.

Intervention (I): Management, treatment, or prevention of dental caries.

Comparison (C): Not applicable.

Comparators were not predefined in the PICOS framework due to substantial heterogeneity in study designs.

Outcomes (O): At least one of the following:

1. Direct medical costs (diagnostic, preventive, restorative, surgical, or emergency care).
2. Direct non-medical costs (transport, food, accommodation, childcare).
3. Indirect costs (school absenteeism, caregiver productivity loss, income loss).
4. Intangible costs (pain, stress, reduced quality of life, QALY/DALY impact).

Study types: Eligible study designs included cost-of-illness studies, full or partial economic evaluations, cross-sectional surveys, retrospective studies, and randomised controlled trials reporting relevant cost data.

Other restrictions: Only studies published in English, in peer-reviewed journals, between January 2012 and July 2025 were eligible for inclusion. The lower date limit was applied to focus the review on contemporary economic evidence that more closely reflects current preventive strategies, treatment pathways, healthcare delivery models, and cost structures. The choice of January 2012 was pragmatic and was not intended to imply that earlier economic evaluations were methodologically invalid. Nevertheless, the date restriction may have excluded relevant earlier studies and was therefore considered a potential source of selection bias.

Studies were excluded if they did not report a relevant economic or cost outcome. Reviews, editorials, opinion articles, case reports, case series, and qualitative studies without quantifiable economic data were also excluded.

### 2.4. Data Collection Process

Data were independently extracted by two reviewers (DG and SJP) using a standardised data extraction form. The reviewers compared their completed forms, and discrepancies were resolved through discussion and consensus. Extracted information included study characteristics, population demographics, intervention and comparator characteristics, cost components, analytical perspective, costing methodology, currency and price year, economic outcomes, and key findings. No attempts were made to contact study authors to obtain or verify missing information, and no automation tools were used during data extraction.

### 2.5. Outcomes and Data Items

The primary outcomes were direct medical costs, direct non-medical costs, indirect costs, and intangible costs associated with the prevention and management of dental caries in individuals under 18 years of age. Additional data extracted included study characteristics, country, study design, population characteristics, intervention and comparator details, study perspective, costing methods, currency, cost-effectiveness outcomes (e.g., QALYs, DALYs, and ICERs), and key findings. All reported results relevant to these outcome domains were extracted where available. When multiple outcome measures or analyses were reported, data considered most relevant to the review objectives were collected.

### 2.6. Other Data Items

In addition to the predefined outcomes, data were extracted on study characteristics (authors, publication year, country, and study design), participant characteristics (age, sex, socioeconomic status, and ethnicity where reported), intervention and comparator characteristics, study perspective, costing methodology, currency, and economic evaluation measures. Funding sources were extracted when reported. No assumptions were made regarding missing or unclear information; only data explicitly reported in the included studies were extracted.

### 2.7. Effect Measures

Because quantitative pooling was not undertaken, no common comparative effect measure was calculated across studies. Results were summarised using the economic measures reported by the original studies, including cost estimates, cost differences, cost savings, QALYs, DALYs, ICERs, and willingness-to-pay estimates, where applicable. Outcomes were summarised descriptively using reported cost estimates (direct, indirect, non-medical, and intangible costs), cost-effectiveness measures (e.g., QALYs, DALYs, and ICERs), and narrative comparisons across studies.

### 2.8. Eligibility for Synthesis

All studies meeting the predefined eligibility criteria and reporting at least one relevant economic outcome were included in the narrative synthesis. No additional eligibility criteria were applied at the synthesis stage.

### 2.9. Data Preparation

No statistical transformations or imputations were performed. Cost estimates were extracted in the currencies, price years, units, and time horizons reported by the original studies. Costs were not converted to a single common currency or reference year because several studies did not report sufficient information regarding price year, inflation adjustment, purchasing power parity, or the underlying costing perspective to support valid and consistent conversion. Consequently, monetary values were interpreted within their original country, healthcare system, price-year, and study perspective contexts rather than compared as directly equivalent estimates.

Where information relevant to an economic outcome was reported in different sections or tables of an included publication, the information was combined to provide a complete description. Missing summary statistics were not imputed.

### 2.10. Synthesis Methods

Quantitative pooling was considered inappropriate because of substantial heterogeneity across the included studies in study design, population, intervention type, healthcare setting, analytical perspective, time horizon, costing methodology, currency, price year, outcome definition, and reporting format. The findings were therefore synthesised narratively.

Studies were first classified by study design, distinguishing trial-based economic evaluations, model-based economic evaluations, and descriptive, observational, or preference-based economic studies. Within these categories, studies were further organised according to intervention type, including population-level preventive strategies, school- or community-based interventions, individual preventive interventions, diagnostic approaches, restorative treatment, and hospital-based care.

Economic findings were summarised according to the reported outcome domain: direct medical costs, direct non-medical costs, indirect costs, intangible consequences, cost-effectiveness, cost-utility, cost–benefit outcomes, QALYs, DALYs, and ICERs. Differences in analytical perspective, time horizon, discounting, currency, price year, healthcare setting, and costing methodology were considered when interpreting similarities and differences across studies.

Study characteristics and economic outcomes were tabulated to support structured cross-study comparison. Heterogeneity was examined descriptively; no pooled estimates, formal subgroup analyses, meta-regression, or review-level sensitivity analyses were performed.

### 2.11. Assessment of Reporting Bias

No formal assessment of risk of bias due to missing results arising from reporting bias (e.g., publication bias) was conducted because a quantitative meta-analysis was not performed and the small number and heterogeneity of included studies precluded meaningful assessment.

### 2.12. Certainty of Evidence

No formal assessment of the certainty (confidence) of the body of evidence, such as the GRADE approach, was conducted because the review consisted of heterogeneous economic evaluations synthesised narratively.

### 2.13. Methodological Quality Assessment

The methodological quality of all included economic evaluations was assessed using the Drummond 10-item checklist [12], which evaluates the clarity of the research question, completeness of alternative comparisons, appropriateness of effectiveness evidence, identification and measurement of costs and consequences, credibility of valuation methods, adjustment for timing, incremental analysis, treatment of uncertainty, and completeness of reporting. Each study was independently assessed by two reviewers (DG and MF) using a standardised scoring approach, with each fulfilled criterion receiving one point (maximum score: 10). Discrepancies between reviewers were resolved through discussion. No automation tools were used during the quality assessment process.

Each applicable criterion was rated as “yes”, “no”, or “unclear”. Criteria rated as “yes” received one point, whereas criteria rated as “no” or “unclear” received zero points. Items considered not applicable because of the study design were excluded from the denominator, and the proportion of applicable criteria fulfilled was calculated for each study.

## 3. Results

### 3.1. Article Selection

The original database searches identified 472 records: 272 from MEDLINE via PubMed and 200 from Scopus. An additional search of Dentistry & Oral Sciences Source (EBSCOhost) conducted during the revision process identified 78 records. After removal of one internal duplicate, 77 records from the additional search were screened against the same predefined eligibility criteria. Two additional reports were assessed at full text, of which one met the inclusion criteria. Overall, 17 studies were included in the systematic review [13,14,15,16,17,18,19,20,21,22,23,24,25,26,27,28,29] (Figure 1).

### 3.2. Risk of Bias

The results of the methodological quality assessment are presented in Table S1. Most included studies fulfilled the majority of applicable Drummond checklist criteria, particularly those relating to the clarity of the research question, identification and valuation of costs and outcomes, and transparent presentation of results [13,15,17,18,19,20,21,22,23,24,25,26].

Several studies fulfilled fewer applicable criteria [14,16,27], most commonly because effectiveness evidence was absent, incremental analysis was not undertaken or was not applicable to the study design, or uncertainty was incompletely addressed. These findings should be interpreted in relation to differences in study design, particularly for descriptive, preference-based, and partial economic evaluations, rather than as evidence of uniformly poor methodological quality.

### 3.3. Main Results

A total of 17 studies were included, representing diverse geographic regions and economic settings. Most studies were conducted in high-income countries, including the United Kingdom and Scotland (*n* = 6) [13,15,19,22,24,29], Australia (*n* = 6) [18,20,21,26,27,28], the United States (*n* = 1) [23], Germany (*n* = 1) [25], and Chile (*n* = 1) [16]. Additional studies originated from China (*n* = 1) [14] and India (*n* = 1) [17].

Study designs included economic modelling studies (*n* = 7) [17,18,22,24,25,29], randomised controlled trials with economic evaluation components (*n* = 5) [13,15,19,23,26], cost-effectiveness and cost-utility analyses (*n* = 4) [16,18,21,25], and population-based or preference studies (*n* = 3) [14,20,27] (Table 1).

The majority of studies focused on children between 0 and 18 years of age, although some modelling studies adopted lifetime horizons to capture the long-term effects of preventive interventions [18]. Gender distribution was generally balanced across study populations. Several studies specifically targeted low-income or socioeconomically disadvantaged groups [14,15,16,18,19,20,24]. Ethnic subgroup analyses were uncommon; however, one US-based study examined differences among Mexican American, non-Hispanic Black, and non-Hispanic White children [22] (Table 1).

### 3.4. Costing and Currency

Costs were reported in the currencies and price years used by the original studies (Table 1). Because the included studies differed substantially in country, healthcare system, analytical perspective, time horizon, resource valuation, currency, and price year, monetary estimates were not treated as directly comparable. Costs were therefore presented in their original reported form. Where original studies had already provided conversions to another currency or purchasing power parity estimates, these values were reported as published and were not recalculated by the review authors.

A UK model-based economic evaluation comparing retention with timely extraction of compromised first permanent molars found that retention generally provided greater long-term tooth-related health benefits, while extraction under general anaesthesia was associated with higher costs [29].

### 3.5. Treatment and Intervention Costs

Direct dental treatment costs varied substantially across countries and intervention types (Table 1).

In high-income countries, average unit costs for common dental procedures ranged from approximately USD 20–30 for fluoride varnish applications, USD 28–120 for fissure sealants, and USD 100–200 for restorative treatments [15,22]. One study reported that hospital-based or sedation-assisted dental procedures were substantially more expensive, reaching up to USD 2777 per case in Medicaid-funded care [22].

In Australia, restoration costs averaged approximately AUD 109–189 per tooth, while preventive interventions such as fluoride varnish cost around AUD 37–55 per application [18,20,21].

Studies conducted in the United Kingdom reported treatment costs ranging from approximately £9–55 for routine procedures, whereas more complex interventions, including extractions under general anaesthesia, reached costs of up to £1165 per case [13,15,19,23,25].

In India and Chile, restorative treatment costs were considerably lower, ranging from approximately USD 12–16 per extraction or filling and around USD 60–65 per child annually, respectively [16,17].

Preventive interventions were consistently associated with lower costs per health outcome than restorative or hospital-based treatments. This pattern was particularly evident for community water fluoridation programmes and school-based toothbrushing interventions, which were repeatedly identified as highly cost-effective approaches for reducing childhood dental caries [13,17,20,26].

### 3.6. Definition of Dental Caries and Intervention Comparisons

Across the included studies, dental caries was consistently described as a preventable oral disease resulting from the demineralization and progressive destruction of dental hard tissues. However, considerable variation was observed in the diagnostic criteria and assessment methods used to identify and measure caries outcomes (Table S2; Supplementary Materials).

Most studies employed standardised clinical assessment tools, including the Decayed, Missing, and Filled Teeth (DMFT/dmft) index and the International Caries Detection and Assessment System (ICDAS), to quantify caries prevalence and severity [20,22,25,26]. Other studies used alternative diagnostic approaches, such as radiographic assessment methods for lesion detection [24] or data obtained through national surveillance systems, including the Public Health England oral health monitoring programme [19]. One model-based economic evaluation considered compromised first permanent molars affected by dental caries or enamel hypomineralization when comparing retention with timely extraction [29].

A smaller number of studies, particularly those conducted in community-based settings or discrete-choice and modelling frameworks, relied on proxy indicators or parent-reported measures of oral health and caries experience [14] (Table S2; Supplementary Materials).

Studies focusing on younger children frequently reported outcomes related to early childhood caries (ECC). In these studies, carious lesions were commonly classified according to lesion depth, severity, or progression beyond the dentino-enamel junction, enabling evaluation of preventive interventions targeting high-risk preschool populations [21] (Table S2; Supplementary Materials).

Interventions evaluated across the included studies varied substantially and included community water fluoridation, supervised toothbrushing programmes, fluoride varnish applications, fissure sealants, behaviour-change interventions, home-visiting programmes, artificial intelligence–assisted diagnostic systems, and broader population-based preventive strategies [13,14,15,16,17,18,19,20,21,22,23,24,25,26,27,28,29]. Comparisons were typically made against usual care, no intervention, alternative preventive programmes, or standard dental service delivery models.

### 3.7. Groups and Interventions Compared

The included studies evaluated a broad range of preventive, diagnostic, and treatment strategies for childhood dental caries. Population-level interventions primarily consisted of community water fluoridation programmes [13,16,26] and fiscal policies aimed at reducing sugar consumption, such as sugar-sweetened beverage taxation [30,31].

School- and community-based interventions included supervised toothbrushing programmes, fluoride varnish applications, oral health promotion activities, and preventive outreach services [17,19,26,27]. Clinical interventions compared alternative treatment or diagnostic approaches, including the Hall Technique versus conventional restorative care [24], retention versus timely extraction of compromised first permanent molars [29], and artificial intelligence (AI)-assisted caries detection systems versus traditional diagnostic methods [25].

Several studies evaluated behavioural and access-based preventive strategies, such as home-visiting programmes, telephone-delivered interventions, and community-based preventive care models [18,21]. Population-level modelling studies also compared risk-based and universal prevention strategies to determine the most efficient allocation of resources [22].

Across studies, preventive and minimally invasive approaches were generally compared with usual care, standard practice, or no intervention. Several evaluations additionally compared different delivery models or intervention intensities, including targeted versus universal prevention and delivery by dental versus non-dental healthcare providers (Table S2; Supplementary Materials).

### 3.8. Direct and Indirect Costs

The direct medical costs reported across studies varied substantially according to intervention type, study perspective, healthcare system, and population size.

In a modelling study of a hypothetical cohort of 50,000 US children aged 1–18 years, total direct costs ranged from approximately USD 61 million under current practice to USD 213 million under a universal prevention strategy, reflecting differences in programme coverage and resource utilisation [22]. Similarly, an Australian modelling study estimated additional programme costs of up to Australian dollar (AUD) 57.5 million for fluoride varnish programmes delivered by dental professionals and Australian dollar (AUD) 23.0 million for home-visiting interventions among 45,677 children from low-income households, highlighting the economic impact of alternative service-delivery models [18] (Table S3; Supplementary Materials).

From a population-health perspective, extending community water fluoridation to Australian communities with at least 1000 residents was estimated to generate approximately Australian dollar (AUD) 95 million in avoided dental treatment costs over a 15-year period while requiring only Australian dollar (AUD) 13 million in implementation expenditures [26]. In contrast, a population-based Australian study reported that hospital admissions related to paediatric oral conditions generated approximately Australian dollar (AUD) 92 million in healthcare expenditure between 2000 and 2009, with nearly half of these costs attributable to dental caries [28].

Indirect and non-medical costs were reported less frequently. One Australian study incorporated indirect costs associated with anaesthesia, pulp therapy, and extractions, estimating additional expenses of approximately Australian dollar (AUD) 90–110 per child [18]. A Chilean Cost–Benefit Analysis conducted from a societal perspective reported reductions in productivity-related costs and human resource expenditures, with water fluoridation reducing absenteeism- and staffing-related costs by approximately 906 CLP per child annually [16] (Table S3; Supplementary Materials).

Two studies specifically quantified infrastructure and system-level implementation costs associated with water fluoridation. One study estimated the total cost of operating a community water fluoridation programme at £2,090,100 over six years, corresponding to approximately £105.63 per child [11]. Another estimated annualised implementation costs of only Australian dollar (AUD) 0.26 per person in urban Australian settings [26].

None of the included studies assigned a monetary value to intangible costs associated with childhood dental caries, such as pain, anxiety, psychosocial burden, or reductions in health-related quality of life.

### 3.9. Individual-Level Costs

At the individual level, direct medical costs varied considerably across countries, intervention types, and time horizons. Studies conducted in the United Kingdom reported annual per-child costs ranging from approximately £28 to £37 [23], whereas an Australian study estimated cumulative treatment costs of approximately USD 1800–3500 per child over a 5.5-year period, reflecting expenditures associated with restorative treatment, general anaesthesia, and repeated episodes of care during early childhood [21].

Preventive interventions were generally associated with lower costs than curative approaches. For example, the Designed to Smile school-based programme in the United Kingdom was estimated to cost approximately £61 per child [19]. Similarly, a fluoride varnish intervention reported average costs of £666 per child in the intervention group compared with £598 under standard care, with staff time accounting for approximately 64% of total programme expenditure [25] (Table S4; Supplementary Materials).

Several studies also quantified indirect and programme-related costs. One study reported modest family-level indirect costs of approximately USD 25–30 per child, primarily attributable to travel expenses and productivity losses [21]. Another study estimated parental productivity losses of approximately £49 per child [25]. Non-medical programme costs, including administration, staff training, educational materials, and implementation expenses, were commonly reported for preventive interventions. For example, a school-based toothbrushing programme reported annual consumable costs of approximately ₹150 (USD 1.9) per child and implementation costs of ₹29,339 (USD 374) over a 12-year period [17].

Intangible costs associated with dental caries, including pain, discomfort, anxiety, and reduced quality of life, were not monetized in any of the included studies. However, several studies incorporated health-related quality of life measures as utility-based proxies to estimate quality-adjusted life years (QALYs), including the Child Health Utility 9D (CHU-9D), EuroQol 5 Dimensions (EQ-5D), Paediatric Quality of Life Inventory (PedsQL), and Scale of Oral Health Outcomes for 5-Year-Old Children (SOHO-5) [25,26] (Table S4; Supplementary Materials).

### 3.10. Cost Calculation Methods and Cost-Effectiveness Outcomes

Cost estimates were primarily generated using economic modelling techniques. The most frequently applied approaches were Markov cohort models [17,18,22,24] and decision-analytic models [20,25], often combined with micro-costing methods or unit-cost estimates derived from national healthcare databases, reimbursement schedules, and published literature. Several studies adopted both healthcare sector and societal perspectives, incorporating caregiver time, productivity losses, and other indirect costs [16,21,22]. Most studies applied annual discount rates ranging from 1.5% to 5%, in accordance with national health economic evaluation guidelines (Table 2).

Per-child costs varied substantially across interventions and settings, ranging from approximately Australian dollar (AUD) 3.9–390 in population-based fluoridation models [26] to approximately USD 1200–6400 in US-based prevention strategies [22]. Preventive interventions, including fluoride varnish programmes, fissure sealants, supervised toothbrushing, and community fluoridation, were consistently associated with favourable economic outcomes, either through cost savings or acceptable incremental cost-effectiveness ratios (ICERs). Two studies reported dominant cost-effectiveness, indicating improved health outcomes at lower overall costs [15,17], while another estimated an ICER of Australian dollar (AUD) 3747 per QALY gained for preventive oral healthcare in an Indigenous Australian community [20].

Quality-adjusted life years (QALYs) were the most commonly reported outcome measure and were derived either from direct utility assessments using instruments such as the CHU-9D, EQ-5D, and PedsQL, or from utility values obtained from the published literature [15,25]. In addition, some studies reported disability-adjusted life years (DALYs) or similar population-health outcome measures, with preventive programmes estimated to avert up to 3700 DALYs through expanded fluoridation and preventive service delivery [18,26] (Table 2).

Overall, ICER estimates demonstrated a consistent trend favouring preventive interventions. Most programmes were either dominant or remained below commonly accepted willingness-to-pay thresholds. Reported ICERs included Australian dollar (AUD) 17,052 per QALY gained for sugar-sweetened beverage taxation [23], USD 83,000–154,000 per QALY gained for population-based prevention strategies in the United States [22], and INR 22,202 per QALY gained (approximately USD 283) for a school-based toothbrushing programme in India [17]. In contrast, some trial-based evaluations reported only marginal differences in QALYs between intervention and control groups and therefore did not meet the National Institute for Health and Care Excellence (NICE) willingness-to-pay threshold of £20,000 per QALY [19,26] (Table 2).

## 4. Discussion

### 4.1. Principal Findings

This systematic review provides a comprehensive synthesis of the economic evidence surrounding the prevention and management of dental caries in children and adolescents under 18 years of age. Across the 17 included studies, substantial variability was observed in costing methods, analytical perspectives, outcome measures, and economic modelling approaches, reflecting differences in healthcare systems, target populations, and methodological frameworks. Despite this heterogeneity, a consistent pattern emerged: preventive interventions were generally more cost-effective than restorative or hospital-based treatments, generating both health gains and economic benefits across diverse settings.

The decision to include studies published from 2012 onwards was intended to capture contemporary economic evaluations conducted within modern healthcare systems and using current methodological standards. Restricting the review to recent evidence improved comparability between studies and enhanced the relevance of findings for current policy and clinical decision-making.

### 4.2. Economic Burden and Cost Drivers

Direct medical costs represented the largest component of the economic burden associated with childhood dental caries across the included studies [15,22,23,25]. Hospital-based dental treatment, particularly procedures performed under general anaesthesia, was consistently identified as one of the most expensive forms of care, with costs frequently exceeding USD 2000 per case in high-income countries [21,22]. The additional model-based evaluation of compromised first permanent molars further demonstrated that treatment strategy can substantially influence long-term costs and tooth-related health outcomes, with retention providing greater quality-adjusted tooth-years (QATYs) than routine extraction in several scenarios [29]. In contrast, preventive interventions such as supervised toothbrushing programmes, fluoride varnish applications, fissure sealants, and community water fluoridation were associated with relatively low per-child costs while generating substantial long-term savings through reductions in restorative treatment, emergency care, and hospital admissions [13,17,20,26].

Indirect and non-medical costs were reported less frequently but contributed meaningfully to the overall economic burden. Studies adopting a societal perspective demonstrated that caregiver productivity losses, travel expenses, and school absenteeism could impose considerable financial consequences on families, particularly those from socioeconomically disadvantaged backgrounds [16,21]. The limited reporting of these cost categories suggests that existing estimates may underestimate the true societal burden of childhood dental caries.

### 4.3. Cost-Effectiveness of Preventive Strategies

Preventive interventions consistently demonstrated favourable cost-effectiveness profiles across a range of healthcare settings. Community water fluoridation, fluoride varnish programmes, school-based toothbrushing initiatives, and other preventive approaches generally produced positive health outcomes at relatively low additional cost [13,17,20,26]. Several interventions were reported as dominant strategies, providing improved outcomes while simultaneously reducing overall costs [15,17].

School-based and home-visiting programmes also generated positive quality-adjusted life year (QALY) gains at comparatively low incremental costs, highlighting their potential as scalable and equitable public health interventions [17,19,20,21]. Although some trial-based evaluations found only small differences in QALYs between intervention and control groups [19,25], these findings likely reflect the relatively short follow-up periods available within clinical trials. Preventive interventions often require many years before their full health and economic benefits become apparent.

In contrast, long-term modelling studies consistently identified prevention as highly cost-effective because they captured reductions in disease progression, restorative treatment needs, and hospital-based care over extended time horizons [18,22,23,26]. The contrast between trial-based and model-based findings is therefore largely explained by differences in analytical time horizons. Preventive interventions typically involve immediate implementation costs, whereas the resulting health gains and cost savings accumulate gradually over time. Decision-analytic models are better able to capture these long-term consequences than short-duration clinical trials.

### 4.4. Indirect, Non-Medical, and Intangible Costs: Evidence Gaps

One of the most important findings of this review is the limited consideration of indirect, non-medical, and intangible costs within existing economic evaluations of childhood dental caries. Although several studies included caregiver productivity losses, travel expenses, and school absenteeism [16,21], these outcomes were measured inconsistently and were often excluded entirely from economic analyses.

Notably, none of the included studies assigned monetary values to intangible consequences such as pain, anxiety, emotional distress, social stigma, or reduced well-being among children and their caregivers. When these impacts were considered, they were typically incorporated indirectly through health-related quality of life instruments used to calculate QALYs rather than through explicit cost valuation. Consequently, the overall societal burden of childhood dental caries may be substantially underestimated.

The absence of robust methods for quantifying intangible costs represents a significant gap in the literature and highlights an important area for future research. More comprehensive economic evaluations incorporating societal perspectives and broader measures of well-being could provide a more accurate estimate of the true burden of childhood dental caries and the value of preventive interventions.

### 4.5. Measurement of Health Outcomes and Intangible Costs

Quality-adjusted life years (QALYs) were the most frequently reported health outcome measure across the included studies and were derived using validated health-related quality of life instruments, including the Child Health Utility 9D (CHU-9D), EuroQol 5 Dimensions (EQ-5D), and Paediatric Quality of Life Inventory (PedsQL) [18,19,22,23,25,26]. QALYs combine both the quantity and quality of life into a single metric, enabling comparisons of cost-effectiveness across different health interventions and disease areas [32,33]. Their widespread use within the included studies reflects their value in informing resource allocation decisions and assessing whether dental interventions provide sufficient health benefits relative to their costs.

Disability-adjusted life years (DALYs), which quantify years of healthy life lost due to disease, disability, or premature mortality, were reported less frequently [34]. DALYs are commonly used in population-health and global-burden analyses because they facilitate comparisons across diseases and health conditions. The inclusion of DALY-based outcomes can therefore support broader public health prioritisation and improve comparisons between oral health programmes and other healthcare interventions.

Despite the recognised importance of psychosocial and quality of life consequences associated with childhood dental caries, few studies explicitly evaluated intangible costs such as pain, anxiety, emotional distress, or social impacts on children and their caregivers [15,16,18]. When these outcomes were considered, they were generally incorporated indirectly through utility measures used to calculate QALYs rather than through direct economic valuation. This finding highlights an important gap in the literature and suggests that current economic evaluations may underestimate the full burden of childhood dental caries.

Only a limited number of studies incorporated DALY-based outcome measures. One population-level evaluation estimated that community water fluoridation could avert approximately 3700 DALYs, demonstrating substantial long-term public health benefits [26]. Expanding the use of DALYs, or combining DALY- and QALY-based approaches, may strengthen future evaluations by improving comparability across countries and supporting integration with broader public health decision-making frameworks.

### 4.6. Geographic and Income-Level Trends

Most included studies were conducted in high-income countries, particularly the United Kingdom, Australia, and the United States [13,15,18,19,20,21,22,23,25,26,27,28]. These countries generally have well-established public dental programmes, comprehensive administrative datasets, and mature frameworks for conducting health economic evaluations. Consequently, much of the available evidence originates from healthcare systems with relatively strong preventive infrastructures and established oral health policies.

Nevertheless, emerging evidence from middle-income countries, including India and China, demonstrates growing recognition of oral health as both a public health and economic priority [14,17]. Findings from these settings suggest that preventive interventions remain cost-effective even in resource-constrained environments where access to dental treatment is limited and financial barriers to care are substantial. These results support the transferability of preventive oral health strategies across a range of economic contexts.

### 4.7. Limitations of the Evidence Base

Several methodological limitations should be considered when interpreting the findings of this review. First, substantial heterogeneity was observed across studies with respect to study design, costing methodology, analytical perspective, discount rates, time horizons, and outcome measures. This variability limited direct comparisons between studies and precluded quantitative synthesis through meta-analysis. Consequently, the findings were synthesised narratively.

Second, studies reported costs using a wide range of currencies, including US dollars (USD), Pound Sterling (£), Australian dollars (AUD), euros (EUR), Chinese yuan (CNY), Indian rupees (INR), and Chilean pesos (CLP). Differences in price years, inflation adjustments, and costing approaches further complicated comparisons of treatment costs and incremental cost-effectiveness ratios across settings. Future reviews may benefit from standardising costs to a common currency and reference year to facilitate cross-country comparisons.

Third, many evaluations relied on modelling assumptions or short-term trial data, which may not fully capture the lifetime costs and benefits associated with preventive dental interventions. Although modelling studies provide valuable long-term projections, their findings are inherently dependent on assumptions regarding disease progression, intervention effectiveness, and healthcare utilisation.

Finally, the exclusion of non-English-language studies may have resulted in the omission of relevant evidence from some regions. In addition, the limited reporting of indirect, non-medical, and intangible costs restricts the ability to estimate the full societal burden of childhood dental caries.

### 4.8. Limitations of the Review Process

This review has several methodological limitations. The electronic search included MEDLINE via PubMed, Scopus, and Dentistry & Oral Sciences Source (EBSCOhost). Although these databases provide complementary coverage of the biomedical, dental, public health, and interdisciplinary health economic literature, specialised economic databases and grey literature sources were not searched systematically. Some relevant unpublished, non-indexed, or specialised economic evaluations may therefore have been missed. However, backward citation searching of included studies and relevant reviews was undertaken to identify additional eligible records.

The restriction to English-language, peer-reviewed studies published from January 2012 onwards may have resulted in language and publication date bias and may have excluded relevant earlier evidence. Accordingly, the review should be interpreted as a synthesis of contemporary rather than historical economic evidence.

In addition, costs were reported across different currencies, price years, healthcare systems, analytical perspectives, and time horizons. Because consistent conversion was not possible for all studies, costs were retained in their original reported form, limiting direct cross-study comparison.

### 4.9. Implications for Policy and Research

The findings of this review indicate that preventive strategies for childhood dental caries generally provide greater economic value than restorative or hospital-based treatment approaches. Community water fluoridation, supervised toothbrushing programmes, fluoride varnish applications, and other preventive interventions consistently demonstrated favourable cost-effectiveness profiles across a range of healthcare settings [13,17,20,26]. These benefits appear particularly important for high-risk populations and socioeconomically disadvantaged communities, where the burden of disease is often greatest.

From a policy perspective, health ministries could use these findings to inform future budget allocation and priority setting for oral health programmes. The evidence supports directing budget to preventive and cost-effective programmes, since increased investment in preventive oral health programmes may reduce future treatment expenditures while simultaneously improving population oral health outcomes. Policymakers should consider integrating preventive dental services into broader public health strategies, including oral health education in kindergartens and schools, supervised toothbrushing programmes in kindergartens, educational programmes for new parents on children’s oral health, and ensuring equitable access to evidence-based preventive interventions.

Future research should prioritise the development of standardised methodologies for measuring indirect and intangible costs, including psychosocial and quality of life impacts on children and their families. Additional long-term economic evaluations conducted in low- and middle-income countries are also needed to strengthen the global evidence base and improve the generalizability of findings across diverse healthcare systems.

## 5. Conclusions

This systematic review demonstrates that dental caries in children under 18 years imposes a substantial economic burden, driven primarily by direct treatment costs, while the review’s results show that preventive interventions are consistently more cost-effective than curative care. However, indirect and intangible costs remain poorly quantified, indicating that the true societal burden of paediatric dental caries is likely underestimated. Future research should prioritise comprehensive costing approaches to better inform policy and resource allocation.

## Figures and Tables

**Table 1:** Characteristics of included studies reporting the cost and cost-effectiveness of dental caries prevention and treatment interventions in children.

Article ID	Type of Study	Country	Ethnicity	Social Economic Status of a Family Where a Child Grows	Age	Sex	Currency	Costs of Actual Dental Treatment
**Janusz et al. [23]**	Cost-effectiveness modelling study.	USA	US population; includes mention of disparities among Mexican American, non-Hispanic Black, and non-Hispanic White children	Low income	Children (1–18 years old)	Both	USD (2021)	Based on Medicaid reimbursement averages (2021–2022) across US states: Fluoride varnish: USD 20.88 per application Dental sealant: USD 28.76 per molar Oral exam: USD 72.66–USD 187.85 depending on age Restoration (fillings/crowns): USD 128.22 average per procedure Sedation (hospital setting): USD 2777 per procedure
**Marshman et al. [19]**	Randomised controlled trial (RCT) with economic evaluation component.	UK	NA	NA	Mean 12.7 years (SD 0.6); range ≈ 11–14 years	Both	Pound Sterling (£)	NA
**Anopa et al. [27]**	Randomised controlled trial (RCT) with economic evaluation—cost-utility (CUA), cost-effectiveness (CEA), and cost-consequence (CCA) analyses.	Scotland	NA	Mostly low-income level	Preschool children aged 3 at baseline	Both	Pound Sterling (£) 2016–2017	Preventive intervention cost only: £32.66 (mean, SD £13.21) per child for the FV arm over 2 years; no restorative or curative treatment costs included
**Koh et al. [21]**	Cost-utility and cost-effectiveness analysis using a Markov cohort model based on data from a randomised controlled trial.	Australia	NA	Low-income level	Children aged 6 months to 6 years.	Both	US dollars (2014), converted from Australian dollars (2013) at a rate of 1.55.	Restoration: USD 83, Crown: USD 219 Extraction: USD 135 General anaesthesia: USD1412, Medication: USD 7 (mean).
**Wang et al. [14]**	Discrete-choice experiment (economic preference study).	China	NA	All levels	3–12 years	Both	Chinese Yuan (CNY, 2023 values)	Fluoride application: 30–60 CNY per child per session Pit and fissure sealant: 80–120 CNY per tooth Oral health education: 10–20 CNY per child
**Schwendicke et al. [24]**	Economic evaluation alongside a randomised controlled split-mouth trial.	Scotland, UK	NA	NA	Children aged 3–10 years (majority 4–9 years)	Both	Pound Sterling (GBP, 2017 values)	Initial costs: Hall Technique GBP 22.85/tooth; Conventional Restoration GBP 8.75/tooth. Total cumulative direct treatment costs: Hall Technique GBP 24.12 (95% CI 23.04–25.24) vs. Conventional Restoration GBP 29.26 (95% CI 17.11–46.42) per tooth. Total cumulative costs including indirect costs: GBP 32.26 vs. GBP 48.91.
**Gomez Rossi et al. [25]**	Economic evaluation using Markov model and Monte Carlo microsimulations (payer perspective).	Germany	NA	All levels	12 years old	Both	Euro (€), converted by Purchasing Power Parity (PPP) to USD 2020 values (€ = USD 1.34)	Per-procedure costs: €40 (exam + X-ray), €150–210 (restoration), €490 (root canal), €70 (extraction), €620 (crown), €1850 (implant).
**Nguyen et al. [18]**	Economic evaluation/cost-effectiveness analysis using Markov model based on existing trial data.	Australia	NA	Low household income	0.5–12 years	Both	Australian dollar (AUD), 2020	Dental check-up: AUD 54.69 ± 3.19 Restoration per tooth: AUD 189.61 ± 17.95 Fluoride varnish (6-monthly): AUD 36.80 × 4 visits Fissure sealant (4 molars): AUD 49.00 each Dental screening: AUD 29.25 Consultation (telehealth): AUD 16.04 Consultation (home visit): AUD 23.33 + travel AUD 12.34
**Victory et al. [15]**	Economic evaluation alongside a pragmatic randomised controlled trial (RCT); decision tree model using NHS perspective.	UK	NA	NA	5–7 years old (children who had at least one primary tooth extracted).	Both	Pound Sterling (£), 2020	(From NHS 2020 price year): Examination (Band 1): £9.40–£29.15 One X-ray: £4.55–£9.65 Fissure sealant: £9.15 Amalgam filling: £9.95–£25.50 White filling: £18.75–£46.74 Root filling (molar): £112.50 Simple extraction: £9.15–£55.25 Surgical extraction: £24.95–£57.25 Dietary advice: £4.48 Oral hygiene instruction: £4.48 Antibiotics: £14.60
**Cobiac et al. [26]**	Cost-effectiveness modelling study using a life-table approach.	Australia	non-Indigenous, nonremote Indigenous, and remote Indigenous populations	All levels	Children (<16 years) modelled primarily	Both	2003 Australian dollars	Derived from Australian Dental Association 2006 rates, adjusted to 2003 AUD: Oral exam: AUD 45 (USD 13) Restoration: AUD 109 (USD 21) OPG radiograph: AUD 82 (USD 15) Average Cost per Caries Case (Base Case) ≈AUD 154 Oral exam + restoration Average Cost per Caries Case (Including X-ray) ≈ AUD 236 Oral exam + restoration + radiograph
**Sharda et al. [17]**	Cost-effectiveness modelling study using a Markov model comparing “no intervention” vs. a supervised toothbrushing (STB) programme.	India	NA	Low socioeconomic level	Hypothetical cohort aged 6–75 years (school-age initiation at 6 years).	Both	Indian Rupees (INR)—converted to US dollars (USD) at an exchange rate of 1 USD = ₹78.4 (2022 values).	Restoration (Filling): ₹1285 (USD 16.4) Endodontic Therapy (Root Canal): ₹7500 (USD 96) Extraction: ₹1000 (USD 12.8) Prosthodontic Replacement: ₹15,000 (USD 191) Consumables (toothbrush, toothpaste, disposables): ₹150 per child per year (USD1.9) Total Lifetime School Program Cost (ages 6–17): ₹29,339 per child (USD 374)
**Kularatna et al. [20]**	Preventive intervention study with 2-year follow-up. (Single preventive intervention of fissure sealant (where indicated), povidone iodine, and fluoride varnish provided to treatment group compared to natural comparison group.) The natural comparison group was like the treatment group except for the extra intervention.	Australia	95%—Indigenous children in Far North Queensland	Low socioeconomic level	6–12-year	Both	AUD (Australian dollars) 2018	Cost of Examination: USD 55 Cost of Restoration: USD 145 Cost of Extraction: USD 195 Cost of Pulp Therapy: USD 77 Cost of Fissure Sealant: USD 47 Cost of Radiography: USD 38 Cost of Varnish: USD 30 Cost of Plaque Removal: USD 55 Cost of Calculus Removal: USD 91 Cost of Oral Hygiene Instructions: USD 50 Cost of Dietary Advice: 37
**Alsaharif et al. [28]**	Population-based cost description study (retrospective analysis using hospital morbidity data).	Australia	Indigenous and non-Indigenous	NA	Children aged 0–14 years.	Both	Australian dollar (AUD)	NA
**Ulloa et al. [16]**	Economic Cost–Benefit Analysis (CBA) adopting a social perspective (including direct and indirect costs).	Chile	NA	Low- to middle-income families	12 years old children	Both	Chilean Peso (CLP USD) and USD (conversion 1 USD = 648.9 CLP), values based on 2017 data.	Without fluoridated water: 42,200 CLP per child (≈USD 65.0) With fluoridated water: 39,051 CLP per child (≈USD 60.2) Savings: 3149 CLP (≈USD 4.9) per child per year.
**Whittaker et al. [13]**	Cost-effectiveness analysis (CEA) with QALY as the outcome measure; prospective longitudinal cohort design.	UK	NA	All levels	Two cohorts: Birth cohort: children followed from birth to 5 years (exposed in utero). Older school cohort: children aged 5–11 years at follow-up.	Both	Pound Sterling (£), 2014	Mean NHS dental cost (per child): Birth cohort: £136.94 (non-fluoridated) vs. £101.27 (fluoridated) → £35.67 savings per child. Older school cohort: £432.73 (non-fluoridated) vs. £360.00 (fluoridated) → £72.73 savings per child. Emergency dental (hospital) cost: minor differences (–£3.89 to –£14.45 per child). Water fluoridation costs: total £2,090,100 (capital + running), or £14.14 per capita (equivalent annualised cost); £105.63 per child aged 0–12 across 6 years.
**Kay et al. [22]**	Cost-Utility Analysis (CUA) using a decision-analytic model comparing community-based tooth brushing and fluoride varnish programmes	UK	NA	Low economic level	5-year-old and 12-year-old children	Both	Pound Sterling (£), 2014	NHS treatment costs estimated from national data: Tooth extraction (under GA)—£1165 per extraction Restoration (filling)—£25 per unit of dental activity, lifetime cost calculated with replacements and inflation adjustment. Future costs are discounted at 1.5% annually. Cost range for treating dental caries used in model: £150–£350 per case (depending on age and treatment type).
**Sanghvi et al. [29]**	Markov model/health economic analysis	United Kingdom	NA	NA	8 years at model entry	NA	GBP (£), 2018/19 prices	Lifetime discounted treatment cost per compromised first permanent molar: £237 for maintenance/retention. Extraction: £9 per cFPM when one tooth was extracted under LA; £631 per cFPM when one tooth was extracted under GA; £316 per cFPM when two cFPMs were extracted under GA; and £158 per cFPM when four cFPMs were extracted under GA.

**Table 2:** Summary of economic evaluation parameters, cost-effectiveness outcomes, and health utility measures (QALY, DALY, ICER) reported in included studies of dental caries prevention and treatment in children.

Article ID	How Were the Costs Calculated?	The Costs of an Individual Calculated	QALY	How QALY Was Calculated	DALY	ICER
**Whittaker et al. [13]**	Included capital + running costs of fluoridation, NHS dental treatment costs (UDA-based), emergency dental care, and general anaesthetic extractions (£935 each); all costs discounted at 3.5%.	Fluoridation cost divided per capita for whole population and per child (0–12 years) using Equivalent Annual Cost (EAC) over 6 years.	Measured using CHU9D utilities.	Area-under-the-curve between baseline and follow-up utilities; discounted 3.5% from year 2.	NA	Fluoridation was dominant.
**Janusz et al. [23]**	Costs were derived by combining: 1. Direct medical costs from state Medicaid fee schedules (2021–22) for preventive and restorative procedures. 2. Caregiver time costs estimated from expert panel input (for societal perspective). 3. Simulation modelling: Aggregated procedure frequencies and costs for a cohort of 50,000 US children aged 1–18 years, using a state-transition (Markov) model built in TreeAge Pro 2022 (Williamstown, MA, USA); the aggregation and cost submodel was programmed in Stata V15 (College Station, TX, USA). 4. Discounting: All costs discounted 3% annually to 2021 values. 5. Perspectives: Both healthcare sector (direct costs only) and limited societal (including caregiver time).	Current practice: USD 61.2 million/50,000 = ~USD 1225 per child Risk-based prevention: USD 146.4 million/50,000 = ~USD 2928 per child Prevention-for-all: USD 213.3 million/50,000 = ~USD 4266 per child From limited societal perspective (including caregiver time): Current practice: USD 98 million/50,000 = ~USD 1960 per child Risk-based: USD 220 million/50,000 = ~USD 4400 per child Prevention-for-all: USD 323 million/50,000 = ~USD 6460 per child	Risk-Based Prevention gained 1030 QALYs vs. current practice (for a cohort of 50,000 children). Prevention-for-All gained an additional 435 QALYs vs. risk-based prevention	0.0085 (0.0049–0.0112) as QALY loss per cycle due to pain due to moderate and severe caries. Discount at 3% annually.	NA	USD 83k/QALY and USD 154k/QALY (health sector)/USD 119k and USD 235k (societal). Risk-based prevention was more cost-effective than universal prevention.
**Marshman et al. [19]**	Based on resource use records (staff hours, materials, school delivery time) and standard UK unit costs from NHS reference costs and educational data	NA	QALY = −0.003 (incremental)	QALYs were estimated using the CHU-9D child utility instrument.	NA	Cost of £340 per QALY, dominated
**Anopa et al. [27]**	Micro-costing from NHS public-sector perspective; unit prices from PSSRU 2017 and NHS Reference Costs 2016/17; discount rate 1.5%; multiple imputation for missing data; GLM adjusted for age, sex, deprivation, baseline utility and caries.	Mean per-child total cost £665.90 (FV) vs. £597.52 (TAU); incremental £68.37 per child (2-year follow-up).	FV = 1.8590 (95% CI 1.8483–1.8674); TAU = 1.8634 (1.8522–1.8729) → Δ = −0.0044 (−0.016 to 0.0069). ICER = −£15 467/QALY (11% probability cost-effective at £20,000 threshold).	QALYs were calculated using CHU-9D (Child Health Utility-9D) instrument. Within-trial utilities collected at baseline, 12 months, and 24 months. Discounted at 1.5% per year (NICE guidelines).	NA	Not cost-effective
**Koh et al. [21]**	From a societal perspective including health-system and parental costs. Costs were discounted at 5% annually and based on Australian Dental Association fee schedules and Pharmaceutical Benefits Scheme prices.	Home-visit intervention saved ≈USD 1670 per 100 children (~USD 16.70 per child) vs. usual care; cost savings per child estimated around USD 1448–USD 1687 depending on sensitivity analysis.	Home-visit group gained 7 QALYs, telephone group 6 QALYs, per 100 children over 5.5 years vs. usual care.	QALYs derived from CHU-9D utility scores, adapted for parental proxy reporting. Used in a Markov model over 5.5 years. Discounted at 5% per year (Australian standard). QALYs = utility × time.	NA	Dominant
**Wang et al. [14]**	Through marginal rate of substitution between service attributes and OOP cost. Values bootstrapped via delta method.	Mean guardian WTP (per child, per service): ≈ 409 CNY for high-effective CPS; typical full preferred package (vacation + high effectiveness + close distance) ≈ 580 CNY per child.	NA	NA	NA	NA
**Schwendicke et al. [24]**	Societal perspective. Direct treatment costs were estimated using NHS Scotland fee schedules (with sensitivity analysis using NHS England fees). Initial treatment, maintenance, retreatment (restorations, pulp therapy, extractions), travel costs, and parental time/opportunity costs were included. Future costs were discounted at 1.5% annually.	Yes. Costs were calculated per tooth (individual treated molar). Direct, indirect, and total cumulative costs were reported per tooth.	NA	NA	NA	Reported as cost-effectiveness using tooth survival outcomes rather than QALYs. Mean ICER = GBP 2.38 spent additionally while losing 1% tooth survival with conventional restoration compared with Hall Technique.
**Gomez Rossi et al. [25]**	Markov model with annual cycles and lifetime horizon; inputs from German public and private fee catalogues; AI charged €8 per radiograph (as fee-for-use); discounted 3%; Monte Carlo (1000 × 1000 simulations).	Per individual (child, 12 y): assuming ~28 teeth × €320 = ≈ €8960 (USD 12,000) lifetime cost with AI vs. €9576 (USD 12,750) ** without AI **—net saving ≈ €616 (USD 820).	For dentistry, the outcome was tooth-retention years ≈ utility proxy; not reported as QALY numerically but 62.4 vs. 60.9 years (+1.5 years gain).	Model-based QALYs from prior studies; lifetime Markov model; Monte Carlo; 3% discount.	NA	Dominant
**Nguyen et al. [18]**	Markov cohort model (TreeAge Pro 2023). Perspective: Australian Government healthcare (NHS equivalent). Costs from government fee schedules, trial data, and Department of Veterans’ Affairs rates. All values in 2020 AUD, discounted 3%. ICERs and uncertainty tested using Monte Carlo simulations (2000 cycles).	Home visits: ≈Australian dollar (AUD) 35/child × 11 visits ≈ AUD 385 per child Telehealth: AUD 16 × 11 = AUD 176 per child Fluoride varnish: ≈AUD 150 per child (4 applications + screening) Fissure sealant: ≈AUD 225 per child (4 molars + screening)	Home visits: +2.4 QALYs Telehealth: +2.8 QALYs Dental screening + fluoride varnish: +28.7 QALYs Fluoride varnish (non-dental): +28.7 QALYs Fissure sealant: +0.7 QALYs	CHU-9D utilities; modelled utility gain (1.0 vs. 0.9 health state); Markov; 2-, 6-, 12-year horizons.	Home visits: 0.4 Telehealth: 0.5 Screening + varnish: 5.0 Non-dental varnish: 5.0 Fissure sealant: 0.1	NA
**Victory et al. [15]**	Derived from NHS payment bands (Bands 1–3) and actual RECUR trial resource use. NHS UDA (Unit of Dental Activity) value: £28. All costs adjusted to 2020 Pound Sterling (£), discounted at 3.5% annually. Incremental cost-effectiveness calculated with Monte Carlo simulation and cost-effectiveness acceptability curve (CEAC).	£115.90 per child (intervention) vs. £119.46 (control) → £3.56 cost saving per child, plus 0.023 QALY gained.	Incremental +0.023 per child for DR-BNI vs. control. Utility: caries-free = 0.96, caries recurrence = 0.88.	QALYs from literature utilities (0.96 vs. 0.88); decision tree; 3.5% discount.	NA	Dominant (range: £119–£2126/QALY in sensitivity)
**Cobiac et al. [26]**	Health sector perspective, 15-year horizon, 3% annual discount rate, costs adjusted to 2003 AUD. Used Monte Carlo simulation and probabilistic sensitivity analysis.	Cost per child: Urban = AUD 3.9 (15 years), Rural = AUD 390 (15 years). Average treatment cost per caries case: AUD 154–236. Programme outcome: Cost-saving—each child yields ~5× return in avoided treatment costs.	NA	NA	DALY (children): 3700 DALYs averted (95% UI: 2200–5700). Disability weight: 0.057 for symptomatic dental caries.	Dominant; AUD 92,000/DALY
**Sharda et al. [17]**	Based on CGHS 2022 rate list + private market survey (Delhi, Haryana, Punjab) 30% public/70% private mix Discount rate: 3% per year Currency: INR 2022, converted at ₹78.4 = US dollar (USD) 1. Lifetime horizon: 6–75 years Markov model cycles: 6 months.	Programme cost per child (12 years): ₹29,339 US dollar 374. Lifetime treatment savings per child: ₹44,874 US dollar (USD) 572. Net lifetime cost saving: ₹15,535 US dollar (USD) 198.	Cost per QALY gained: ₹12,815 US dollar (USD) 164 (public sector scenario) ₹22,202, US dollar (USD) 283 saved per QALY (real-world scenario).	QALYs from EQ-5D-5L utilities (Indian tariff), multiplied over 12 months.	NA	INR 22,202/QALY (~US dollar (USD) 283), Dominant
**Kularatna et al. [20]**	The mean cost per treated child over a 10-year period using expected cost analysis. Economic model used-Markov modelling	Expected-cost analysis using a 10-year Markov model. Treatment costs were obtained from the Queensland Health Department fee schedule and reported in 2018 AUD. Costs included examination, radiography, restorations, extractions, pulp therapy, fissure sealants, fluoride varnish, plaque/calculus removal, oral hygiene instruction, and dietary advice. Costs and utilities were discounted at 5% annually.	For a cohort of 500 children over 10 years: intervention = AUD 508,500 (≈AUD 1017/child); comparison = AUD 175,500 (≈AUD 351/child). Incremental cost = AUD 333,000 (≈AUD 666/child).	Intervention: 4625 QALYs; comparison: 4535 QALYs; incremental gain: 90 QALYs per cohort of 500 children over 10 years.	QALYs were derived from CHU−9D utility values. Utility for active caries = 0.87 in the base case and utility for health = 1.00. Utilities were incorporated into the 10-year Markov model and discounted at 5% annually.	QALYs from CHU-9D utilities (Australian tariffs); 10-year Markov model
**Alsaharif et al. [28]**	Data source: Hospital Morbidity Data System (HMDS), Western Australia (2000–2009). Costing method: Each hospitalisation episode was assigned a Diagnosis-Related Group (DRG) cost from the WA Department of Health database. Direct costs: Operating theatre, anaesthesia, and ward costs associated with dental procedures. Indirect costs: Estimated by applying a 1.5× multiplier to total direct costs (reflecting lost productivity and additional societal burden). Inflation adjustment: All values reported in constant AUD (inflation-corrected). Perspective: Health system with an extended estimate to a societal perspective (via the 1.5× multiplier).	Mean hospital cost per child: AUD 19,975 (based on DRG average). Estimated total economic cost per child (including indirect): ≈AUD 30,000 (using 1.5× multiplier). Caries-related cases: ~50% of admissions, thus per-child cost for caries ≈ AUD 10,000 direct + AUD 5000 indirect.	NA	NA	NA	NA
**Ulloa et al. [16]**	Perspective: Societal (included direct medical, indirect, and programme costs). Data sources: Official cost data from FONASA (Chilean National Health Fund) and the Biobío Regional Health Service. -Method: Compared two scenarios (with and without fluoridation) over one year for 23,014 children aged 12. -Cost components: Professional time, supplies, facility costs, transportation, absenteeism. -Currency: 2017 Chilean Pesos (CLP), converted to USD (1 USD = 648.9 CLP). -Discounting: Not applied (1-year horizon).	Per-child cost of dental care: Without fluoridation: 42,200 CLP (≈USD 65). With fluoridation: 39,051 CLP (≈USD 60). Cost of water fluoridation programme: ≈76–170 CLP per child (USD 0.12–0.26).	NA	NA	NA	NA
**Sanghvi et al. [29]**	Markov model estimating lifetime treatment costs over 62 years. Costs were primarily derived from UK NHS sources (Scottish Statement of Dental Remuneration 2019, Northern Ireland Statement of Dental Remuneration 2018–19, and NHS Improvement National Cost Collection), supplemented by average company prices and expert opinion. Costs and outcomes were discounted at 3.5% annually.	Costs were calculated per compromised first permanent molar (cFPM) for maintenance/retention versus extraction strategies.	NA	NA	NA	Maintenance vs. extraction: approximately £99/QATY when four cFPMs were extracted under GA and £34/QATY when one cFPM was extracted under LA. For one or two cFPMs extracted under GA, extraction was dominated (more costly and less effective than maintenance), therefore an ICER was not reported.

## Data Availability

The original contributions presented in this study are included in the article and Supplementary Materials. Further inquiries can be directed to the corresponding authors.

## Supplementary Materials

The following supporting information can be downloaded at https://www.mdpi.com/article/10.3390/dj14090578/s1. Table S1: Methodological quality assessment of the included studies using the Drummond 10-item checklist; Table S2: Definitions of dental caries and characteristics of interventions and comparators across the included studies; Table S3: Summary of direct medical, direct non-medical, and indirect costs reported in the included studies; Table S4: Individual-level costs and health-related quality-of-life outcomes reported in the included studies; Table S5: Complete database-specific search strategies, search dates, limits, and numbers of records retrieved; Supplementary File S1: PRISMA 2020 checklist.

## Abbreviations

AI	Artificial Intelligence
AUD	Australian Dollar
CBA	Cost–Benefit Analysis
CEA	Cost-Effectiveness Analysis
CHEERS	Consolidated Health Economic Evaluation Reporting Standards
CHU-9D	Child Health Utility 9 Dimensions
CLP	Chilean Peso
CNY	Chinese Yuan
DALY	Disability-Adjusted Life Year
DMFT	Decayed, Missing, and Filled Teeth
dmft	Decayed, Missing, and Filled Primary Teeth
DRG	Diagnosis-Related Group
ECC	Early Childhood Caries
EQ-5D	EuroQol 5 Dimensions
FV	Fluoride Varnish
GA	General Anaesthesia
GBD	Global Burden of Disease
HMDS	Hospital Morbidity Data System
ICDAS	International Caries Detection and Assessment System
ICER	Incremental Cost-Effectiveness Ratio
INR	Indian Rupee
MeSH	Medical Subject Headings
NHS	National Health Service
OPG	Orthopantomogram (Panoramic Dental Radiograph)
PICOS	Population, Intervention, Comparison, Outcomes, Study Design
PedsQL	Paediatric Quality of Life Inventory
PPP	Purchasing Power Parity
PRISMA	Preferred Reporting Items for Systematic Reviews and Meta-Analyses
PROSPERO	International Prospective Register of Systematic Reviews
PSSRU	Personal Social Services Research Unit
QALY	Quality-Adjusted Life Year
RCT	Randomised Controlled Trial
SOHO-5	Scale of Oral Health Outcomes for 5-Year-Old Children
STB	Supervised Toothbrushing
UDA	Unit of Dental Activity
USD	United States Dollar
WHO	World Health Organization
WTP	Willingness to Pay
