# Supplementary material for: Direct, Indirect, and Intangible Costs in the Management of Dental Caries in Children Under 18: A Systematic Review
Source: Dent J (Basel). 2026 Sep 9;14(9):578. doi: 10.3390/dj14090578 (PMC13605095; doi:10.3390/dj14090578)
Supplement: Supplementary file 1 [file dentistry-14-00578-s001.zip › dentistry-4413969-supplementary.pdf]

Table S1. Quality assessment of included economic evaluations using the Drummond 10-item checklist.

Study	Research question well defined?	Comprehensive description of alternatives?	Effectiveness of program established?	Important & relevant costs & consequences for each alternative identified?	Costs & consequences measured accurately & appropriately?	Costs & consequences valued credibly?	Costs & consequences adjusted for differential timing?	Incremental analysis of costs & consequences performed?	Allowance made for uncertainty in estimates?	Presentation & discussion of study results include all issues of concern to users?
**Whittaker et al. [13]**	+	+	+	+	+	+	+	+	+	+
**Wang et al. [14]**	+	+	NA	+	+	+	NA	NA	+	+
**Victory et al.[15]**	+	+	+	+	+	+	+	+	+	+
**Ulloa et al.[16]**	+	+	+	+	+	+	NA	+	-	+
**Sharda et al.[17]**	+	+	+	+	+	+	+	+	+	+
**Nguyen et al.[18]**	+	+	+	+	+	+	+	+	+	+
**Marshman et al.[19]**	+	+	+	+	+	+	+	+	+	+
**Kularatna et al.[20]**	+	+	+	+	+	+	+	+	+	+
**Koh et al.[21]**	+	+	+	+	+	+	+	+	+	+
**Kay et al.[22]**	+	+	+	+	+	+	+	+	+	+
**Janusz et al.[23]**	+	+	+	+	+	+	+	+	+	+
**Schwendicke, et al. [24]**	+	+	+	+	+	+	+	+	+	+
**Gomez Rossi et al.[25]**	+	+	+	+	+	+	+	+	+	+

**Cobiac et al.[26]**	+	+	+	+	+	+	+	+	+	+
**Anopa et al.[27]**	+	+	+	+	+	+	+	+	+	+
**Alsharif et al.[28]**	+	NA	NA	+	+	+	NA	NA	-	+
**Sanghvi et al.[29]**	+	+	+	+	+	+	+	+	+	+

Table S2. Characteristics of included studies: disease definitions and intervention groups compared in economic evaluations of dental caries prevention and treatment in children.

Article ID	Disease	Definition of dental caries	Groups compared
**Janusz et al. [23]**	Dental caries	A preventable oral disease characterized by the demineralization and destruction of tooth enamel; modeled using the International Caries Detection and Assessment System (ICDAS) categories of initial, moderate, and extensive lesions	Current practice (limited prevention coverage) vs. Risk-based prevention (targeted to high-risk children using Caries Risk Assessment) vs. Prevention-for-all (universal prevention for all children).
**Marshman et al. [19]**	Dental caries (early to moderate decay)	Clinical dental examination in schools by calibrated examiners following Public Health England surveillance criteria. Caries recorded at the tooth level as decayed, missing, or filled (DMFT/dmft index). Decay defined as cavitation, undermined enamel, or softened dentine visible to the naked eye. Only cavitated and treated lesions counted.	Designed to Smile (D2S) national child oral health improvement programme - supervised toothbrushing and targeted fluoride varnish applications in schools and nurseries - vs. no intervention (pre-programme baseline).
**Anopa et al. [27]**	Dental caries in deciduous teeth	Decayed, missing, and filled teeth ($d_3$mft index) assessed by trained and calibrated examiners following WHO epidemiological criteria for visible lesions (cavitated lesions only)	Fluoride varnish + Childsmile (TAU) vs. Childsmile only (TAU), twice-yearly FV application in nursery schools compared with standard Childsmile care.
**Koh et al. [21]**	Early Childhood Caries (ECC)	A tooth classified as “decayed” if the lesion progressed beyond the dentino-enamel junction and involved dentine; white-spot lesions were excluded.	Home-visit program vs. Telephone-contact program vs. Usual care (no early preventive intervention), early childhood caries prevention delivered by oral health therapists.
**Wang et al. [14]**	Dental caries (prevention in school-aged children)	Conceptual/WHO definition, “decay of tooth hard tissues caused by bacteria.” Assessment method: Parent-reported history, no clinical measure (no DMFT, no ICDAS) used.	School-based oral-health education + biannual fluoride-varnish programme vs. usual care (no fluoride-varnish).
**Schwendicke et al. [24]**	Dental caries	Cavitated dentinal carious lesions in primary molars	Hall Technique vs Conventional Restoration
**Gomez Rossi et al. [25]**	Dental caries	Radiographically detected proximal caries lesions classified as E1-2 (micro-invasive) and D1-3 (invasive) per Schwendicke et al. 2015 and Cantu et al. 2020 (radiographic depth criteria).	AI-assisted radiographic caries detection (CNN support) vs. standard dentist-performed caries detection without AI.
**Nguyen et al. [18]**	Dental caries (decay in deciduous and permanent teeth)	Defined using DMFT/dmft (Decayed, Missing, and Filled Teeth Index), a proxy measure for caries experience, reflecting decayed, missing, or filled teeth. Dental caries incidence derived as change in DMFT.	Five preventive strategies (anticipatory guidance via home visits or telehealth; school-based fluoride varnish by dental or non-dental professionals; fissure sealant program) vs. no intervention (status quo).

**Victory et al. [15]**	Dental caries recurrence in children	Definition of dental caries: Recurrence of decay requiring a new filling or extraction (identified via NHS treatment records). No standardized diagnostic index (e.g., DMFT or ICDAS) was applied.	School-based comprehensive caries prevention program (screening, sealants, varnish, education) vs. usual care (no program).
**Cobiac et al., [26]**	Dental caries	Based on DMFT/dmft index (Decayed, Missing, Filled Teeth); disability weight 0.057 for symptomatic dental caries (from Australian Burden of Disease study).	Seven population-level caries prevention interventions (water fluoridation, fluoride varnish, fissure sealants, fluoride toothpaste promotion, milk and salt fluoridation, and combination strategies) vs. no intervention.
**Sharda et al., [17]**	Dental caries	Based on National Oral Health Survey (NOHS) data using DMFT index (Decayed, Missing, Filled Teeth). A "carious tooth" = decayed tooth requiring restorative treatment or extraction.	Comprehensive school-based oral health promotion (toothbrushing, fluoride varnish, and education) vs. no school-based programme (usual care).
**Kularatna et al. [20]**	Dental caries	No Active: Children with ICDASII Codes 0,1,2 Or After completion of treatment for ICDAS II codes3,4,5,6<br>Active: Children with ICDAS II Codes 3,4,5,6<br>Untreated: Children who did not seek or get treatment	Annual professional preventive intervention (fissure sealants, fluoride varnish, hygiene and diet education) vs. usual reactive care (no structured prevention) in remote Indigenous communities.
**Alsaharif et al. [28]**	Dental caries and other oral conditions requiring hospitalization (embedded/impacted teeth, pulp/periapical diseases, birth/developmental defects, fractures, dentofacial anomalies).	Classified by ICD-10-AM diagnosis codes (International Classification of Diseases - Australian Modification). Dental caries identified under K02 codes.	School-based oral health education + supervised toothbrushing + fluoride varnish vs. no structured programme (usual care).
**Ulloa et al. [16]**	Dental caries	Assessed using DMFT index (decayed, missing, filled teeth), the standard WHO indicator for caries experience in children.	Community-based fluoride varnish, supervised brushing, and education vs. usual care (no organized prevention).
**Whittaker et al. [13]**	Dental caries	Measured by dmft (decayed, missing, filled teeth) index and presence of decay (yes/no). Clinical outcomes included volume of dmft avoided and proportion of decay-free children.	Community water fluoridation vs. no fluoridation - population-level comparison of caries prevention and QALYs gained.
**Kay et al. [22]**	Dental caries	Measured as dmft/DMFT > 0 (decayed, missing, and filled teeth); reflects the presence of any dental decay. Disease states included decayed, restored, or extracted teeth due to caries.	Structured dental health education by dental care professionals vs. usual opportunistic advice (routine care).

**Sanghvi et al. [29]**	Compromised first permanent molars (cFPMs) affected by dental caries or enamel hypomineralization	Compromised first permanent molars affected by dental caries and/or enamel hypomineralization requiring management. The economic model included children aged 8 years with one or more cFPMs.	Extraction strategy: timely removal of the cFPM(s), facilitating spontaneous space closure vs. Maintenance strategy: retention of the cFPM(s) with restorative treatment and subsequent management as required.

Table S3. Summary of cost components reported in included economic evaluations of dental caries prevention and treatment in children.

Article ID	Total direct medical costs	Non-direct medical costs	Non-medical costs	Intangible costs
**Janusz et al.[23]**	Current practice: USD 61 million<br>Risk-Based Prevention: USD 146 million<br>Prevention for All: USD 213 million	NA	NA	NA
**Nguyen et al.[18]**	Base-case scenario:<br>Home visits:<br>+AUD 23.0 million<br>Telehealth:<br>+AUD 18.5 million<br>Fluoride varnish (dental practitioners):<br>+AUD 57.5 million<br>Fluoride varnish (non-dental health professionals): +AUD 34.7 million<br>Fissure sealant:<br>+AUD 10.5 million	Non-direct medical costs (children): GA = AUD 3,074 per case;<br>pulp therapy = AUD 235; extraction = AUD 156 (public) / 550 (private). Weighted 15% : 85%. Added ≈ AUD 90–110 per child in sensitivity analysis.	NA	NA
**Cobiac et al.[26]**	Included dental treatment costs for restorations, exams, and radiographs.<br>Total treatment cost offset: AUD 95 million (range AUD 45–170 million) saved.	Included capital and operational costs of water fluoridation systems.<br>Urban cost:<br>AUD 0.26/person/year (±0.05)<br>Rural cost:<br>AUD 26/person/year (±5)<br>Total implementation cost: AUD 13 million (range AUD 8.6–18 million).	NA	NA
**Kularatna et al.[20]**	Treatment group: AUD 508 500<br>Comparison group: AUD 175 500	NA	NA	NA
**Alsaharif et al.[28]**	AUD 92 million (2000–2009) - total hospital treatment cost for oral conditions in children aged 0–14 years, derived from Diagnosis-Related Group (DRG) hospital data.<br>Dental caries alone:<br>AUD 45 million (≈ 49% of total costs).<br>Mean cost per hospitalization:<br>AUD 19,975.	Estimated at AUD 138 million	NA	NA

**Ulloa et al.[16]**	Without fluoridation:<br>CLP 971,189,189 (≈ USD 1,497,000).<br>With fluoridation:<br>CLP 898,709,139 (≈ USD 1,385,000).<br>Total savings:<br>CLP 72,480,050 (≈ USD 112,000)	Human resources (staff costs):<br>Without fluoridation:<br>CLP 25,729 per child<br>With fluoridation:<br>CLP 24,029 per child<br>Savings:<br>CLP 1,701 per child (≈ USD 2.6).	Transport savings: CLP 329 per child. Absenteeism savings: CLP 577 per child. Total indirect non-medical savings: CLP 906 per child (≈ USD 1.4).	NA
**Whittaker et al.[13]**	Direct medical cost savings:<br>£163,000 (birth cohort) and £395,000 (older cohort) over 6 years.	Total fluoridation system cost:<br>£2,090,100 (capital + running, over 6 years).<br>Equivalent annual cost: £14.14 per capita, or £105.63 per child (0–12 years).	NA	NA
**Sanghvi et al.[29]**	Maintenance/retention: £237 per cFPM. Extraction: £9 per cFPM for one cFPM under LA; £631 per cFPM for one cFPM under GA; £316 per cFPM for two cFPMs under GA; £158 per cFPM for four cFPMs under GA. Costs included restorative treatment, extraction, GA, endodontic treatment, prosthetic treatment, implants, orthodontic treatment and other subsequent dental care. All costs reported in 2018/19 GBP.	NA	NA	NA

Table S4. Individual-level cost components reported in included economic evaluations of dental caries prevention and treatment in children.

Article ID	Direct medical costs (per person)	Non-direct medical costs	Non-medical costs	Intangible costs
**Marshman et al/ [19]**	Direct medical costs (program): Total GBP 121,970 → ≈ GBP 61 per child (includes staff GBP 63,540; materials 21,380; training 9,420; admin 27,630).	NA	NA	NA
**Koh et al.[21]**	Direct medical costs were the largest component, estimated at approximately USD 1,650-1,850 per child, or about USD 165,000-185,000 per 100 children. These included expenses for extractions, general anesthesia, restorations, crowns, and medications.	Non-direct medical costs (indirect costs to families) were much smaller, averaging around USD 25-30 per child, or 2,500-3,000 per 100 children. These covered travel expenses, parental loss of income, and over-the-counter medications.	Ranged from USD 14-32 per child, or 1,400-3,200 per 100 children. These included the costs of staff time, vehicle transport for home visits, administration, and educational materials such as toothbrush and toothpaste packs.	NA
**Wang et al.[14]**	Total direct medical costs: 10-100 CNY per tooth (model levels)	NA	NA	NA
**Schwendicke et al.[24]**	Initial treatment costs, maintenance costs, and retreatment costs (rerestorations, pulp therapy/endodontic treatment, extractions). NHS Scotland perspective: cumulative direct dental treatment costs GBP 24.12 (HT) vs GBP 29.26 (CR) per tooth.	NA	Included. Travel costs and parental time/opportunity costs were calculated. Travel: GBP 5.00 (HT) vs GBP 10.29 (CR). Time costs: GBP 3.21 (HT) vs GBP 8.83 (CR).	NA
**Anopa et al. [27]**	Total mean per-child cost (FV group) £665.90 (SD 70.74) vs control £597.52; incremental Pound Sterling 68.37 (95 % CI – £18.04 to £143.82); main cost drivers = staff labor 64% (£7.59 per visit), disposables 20% (£2.32), travel 15% (£1.78), reusables 1%(£0.08)	Non-direct medical costs (societal perspective, SA6): FV £ 112.54 vs TAU Pound Sterling 73.05 → Incremental ≈ Pound Sterling 39-54 per child (mainly due to parental productivity loss ≈ Pound Sterling 49.38).	NA	Not monetized. Assessed through utility and quality-of-life instruments (CHU9D, PedsQL, SOHO-5).
**Gomez Rossi et al.[25]**	Total direct medical cost per child: ≈ € 8 960 (AI) vs € 9 576 (standard) → € 616 saved per child over lifetime.	NA	NA	NA

**Victory et al.[15]**	£115.90 (intervention) and £119.46 (control) per child, calculated from NHS dental treatment data (fillings, extractions, sealants, varnish, etc.). Intervention cost itself: £6.47 per child.	NA	NA	NA
**Sharda et al.[17]**	Lifetime Dental Treatment Savings: ₹44,874 per child (USD 572) Program implementation cost: ₹29,339 (USD 374) per child (over 12 school years).	Human resources: ₹10,000 (USD 128) per school/year Training & logistics: ₹5,000 (USD 64) per school/year Overheads (administration, electricity, etc.): ₹1,200 (USD 15) per child Consumables (toothbrush, toothpaste): ₹150 (USD1.9) per child/year.	NA	NA
**Kay et al.[22]**	The total direct medical costs were not reported as a single total figure, but they can be derived from the parameters provided in the model.<br><br>Total Direct Medical Costs (modeled NHS expenditure): £59,000–£149,000 per 1,000 children (£150–£350 per caries case)	Preventive intervention costs (public health spending rather than treatment):<br>– Supervised tooth brushing programs.<br>– Fluoride varnish applications.<br>These were modeled as program costs to local authorities, not direct NHS clinical expenses.<br>Patient charges for dental care were excluded (analysis used only public sector/NHS perspective).	NA	Represented by QoL
**Sanghvi et al.[29]**	Not reported per person. Costs were modelled per compromised first permanent molar (cFPM): maintenance/retention £237 per cFPM; extraction costs varied according to number of cFPMs extracted and use of LA/GA.	NA	NA	NA

Table S5. Complete database-specific search strategies.

Database	Platform	Search date	Search strategy	Limits	Records retrieved
MEDLINE	PubMed	July 22, 2025	("Dental caries"[Mesh Terms] OR "dental decay" OR "tooth decay" OR "caries") AND ("cost of illness" OR "economic burden" OR "medical costs" OR "non-medical costs" OR "indirect costs" OR "intangible costs" OR "out-of-pocket" OR "school absenteeism" OR "productivity loss" OR "caregiver burden" OR "quality-adjusted life years" OR "QALY" OR "DALY")	English language; publication period: January 2012–July 2025; peer-reviewed publications	272
Scopus	Elsevier	July 22, 2025	TITLE-ABS-KEY(("dental caries" OR "tooth decay") AND ("cost of illness" OR "economic burden" OR "medical costs" OR "non-medical costs" OR "indirect costs" OR "intangible costs" OR "school absenteeism" OR "QALY" OR "DALY"))	English language; publication period: January 2012–July 2025; peer-reviewed publications	200
Dentistry & Oral Sciences Source	EBSCOhost	19 August, 2026	("dental caries" OR "dental decay" OR "tooth decay" OR caries) AND ("cost of illness" OR "economic burden" OR "medical costs" OR "non-medical costs" OR "indirect costs" OR "intangible costs" OR "out-of-pocket" OR "school absenteeism" OR "productivity loss" OR "caregiver burden" OR "quality-adjusted life years" OR QALY OR DALY)	English; peer-reviewed; January 2012-July 2025	78
